# Evaluation of rK39 Rapid Diagnostic Tests for Canine Visceral Leishmaniasis: Longitudinal Study and Meta-Analysis

**DOI:** 10.1371/journal.pntd.0001992

**Published:** 2013-01-10

**Authors:** Rupert J. Quinnell, Connor Carson, Richard Reithinger, Lourdes M. Garcez, Orin Courtenay

**Affiliations:** 1 School of Biology, University of Leeds, Leeds, United Kingdom; 2 School of Life Sciences, University of Warwick, Coventry, United Kingdom; 3 London School of Hygiene and Tropical Medicine, London, United Kingdom; 4 School of Public Health and Health Sciences, George Washington University, Washington, D. C., United States of America; 5 RTI International, Washington, D. C., United States of America; 6 Instituto Evandro Chagas, Belém, Pará, Brazil; Barcelona Centre for International Health Research (CRESIB), Spain

## Abstract

**Background:**

There is a need for sensitive and specific rapid diagnostic tests (RDT) for canine visceral leishmaniasis. The aims of this study were to evaluate the diagnostic performance of immunochromatographic dipstick RDTs using rK39 antigen for canine visceral leishmaniasis by (i) investigating the sensitivity of RDTs to detect infection, disease and infectiousness in a longitudinal cohort study of natural infection in Brazil, and (ii) using meta-analysis to estimate the sensitivity and specificity of RDTs from published studies.

**Methodology:**

We used a rK39 RDT (Kalazar Detect Canine Rapid Test; Inbios) to test sera collected from 54 sentinel dogs exposed to natural infection in an endemic area of Brazil. Dogs were sampled bimonthly for up to 27 months, and rK39 results compared to those of crude antigen ELISA, PCR, clinical status and infectiousness to sandflies. We then searched MEDLINE and Web of Knowledge (1993–2011) for original studies evaluating the performance of rK39 RDTs in dogs. Meta-analysis of sensitivity and specificity was performed using bivariate mixed effects models.

**Principal Findings:**

The sensitivity of the rK39 RDT in Brazil to detect infection, disease and infectiousness was 46%, 77% and 78% respectively. Sensitivity increased with time since infection, antibody titre, parasite load, clinical score and infectiousness. Sixteen studies met the inclusion criteria for meta-analysis. The combined sensitivity of rK39 RDTs was 86.7% (95% CI: 76.9–92.8%) to detect clinical disease and 59.3% (37.9–77.6%) to detect infection. Combined specificity was 98.7% (89.5–99.9%). Both sensitivity and specificity varied considerably between studies.

**Conclusion:**

The diagnostic performance of rK39 RDTs is reasonable for confirmation of infection in suspected clinical cases, but the sensitivity to detect infected dogs is too low for large-scale epidemiological studies and operational control programmes.

## Author Summary

Canine visceral leishmaniasis is a vector-borne disease caused by the intracellular parasite *Leishmania infantum*. It is an important veterinary disease, and dogs are also the main animal reservoir for human infection. The disease is widespread in the Mediterranean area, and parts of Asia and South and Central America, and is potentially fatal in both dogs and humans unless treated. Diagnosis of canine infections requires serological or molecular tests. Detection of infection in dogs is important prior to treatment, and in epidemiological studies and control programmes, and a sensitive and specific rapid diagnostic test would be very useful. Rapid diagnostic tests (RDTs) have been developed, but their diagnostic performance has been reported to be variable. We evaluated the sensitivity of a RDT based on serological detection of the rK39 antigen in a cohort of naturally infected Brazilian dogs. The sensitivity of the test to detect infection was relatively low, but increased with time since infection and the severity of infection. We then carried out a meta-analysis of published studies of rK39 RDTs, evaluating the sensitivity to detect disease and infection. The results suggest that rK39 RDTs may be useful in a veterinary clinical setting, but the sensitivity to detect infection is too low for operational control programmes.

## Introduction

Zoonotic visceral leishmaniasis (ZVL) is a potentially fatal disease caused by the intracellular protozoan parasite *Leishmania infantum*, which is endemic in South and Central America, the Mediterranean basin and parts of Asia. The domestic dog is the most important reservoir host, and infection is maintained by transmission between dogs by phlebotomine sandfly species [1]. Control of ZVL focuses on the detection and elimination of infected dogs (particularly in Latin America), control of vectors and human case detection and treatment. There is little evidence for the effectiveness of dog culling [1]–[3]. Treatment of infected dogs is also not an effective control method, due to the cost and long course of treatment, and frequent relapse [4]. Canine treatment using human therapeuticals will also increase the risk of drug resistance, and has been banned in some endemic countries. The use of long-lasting insecticides, applied to dogs as collars, spot-on formulations or baths, is a potential control strategy; such methods have not yet been widely tested or implemented, though are increasingly purchased commercially for protection of individual dogs [1], [2], [5].

Diagnosis of ZVL is usually carried out using serological tests based on crude or recombinant parasite antigens, or molecular tests such as PCR, since parasitological diagnosis has low sensitivity. Serological tests such as ELISA and DAT generally have high sensitivity, while the sensitivity of IFAT is usually lower; specificity of serology is more variable [2]. PCR, particularly quantitative real-time PCR, can be more sensitive than serology, and has high specificity [6], [7]. Current guidelines for the diagnosis of ZVL suggest the use of quantitative serology followed by PCR [8], and this combination allows the detection of most infected dogs. However, these tests have important drawbacks for operational control programmes in resource-poor countries: testing requires technical skills and adequate sample storage prior to testing, and transport and laboratory processing leads to time delays between sampling, diagnosis, and the application of the control intervention. Both theoretical and empirical studies have highlighted delays between sampling and culling, coupled with the lack of sensitivity of some serological tests, as important factors underlying the failure of dog culling to control infection in Brazil [3], [9]. In an operational setting, a rapid diagnostic test (RDT) with high sensitivity that would allow for timely *in situ* diagnosis of infection would thus be invaluable for large scale control of infected dogs [2]. In a clinical setting, RDTs would be useful to confirm diagnosis of canine leishmaniasis, as clinical signs are not necessarily specific to ZVL. The diagnostic performances required for these two settings are very different: for veterinarians, high sensitivity and high specificity in the diagnosis of clinical disease is critical, while for use in control programmes, high sensitivity to detect infected and infectious dogs is more important.

Several RDTs have now been developed for the diagnosis of VL in both humans and dogs. The most widely used are immunochromatographic dipstick tests based on the *Leishmania* rK39 antigen. rK39 is a 39 amino acid repetitive immunodominant B-cell epitope in a kinesin-related protein, which is conserved between *L. infantum* and *L. donovani*, and provides high sensitivity to detect clinical cases in ELISA [10], [11]. Incorporation of this antigen into a dipstick format has the major advantages of increased ease and speed of use, with a diagnostic result indicated within minutes without the need for specialist equipment. A number of studies have investigated the use of rK39 RDTs for the diagnosis of human VL, and a recent meta-analysis concluded that rK39 RDTs had high sensitivity (93.9%) and specificity (90.6%) to detect clinically symptomatic human cases [12]. Individual studies on dogs using RDTs show a variable sensitivity and specificity [13]–[28], with no published meta-analysis study to estimate cross-study performance. Sensitivity has been reported to vary with clinical status [18], [22] and antibody titre [16], [21], but little information is available on other variables that potentially affect rK39 positivity. In particular, variation in sensitivity during the course of natural infection has not been examined: studies to date have been mostly cross-sectional, with longitudinal data limited to two small studies [29], [30]. Moreover, research to measure the association between rK39 RDT positivity and canine infectiousness to sandflies has not yet been carried out. Since infectious rather than infected dogs are the epidemiologically important target to reduce ZVL transmission, performance of diagnostic tests to detect infectious dogs is a research priority [2].

The aims of the current study were (i) to evaluate the sensitivity of a rK39 RDT to detect infection, disease and infectiousness in a cohort of naturally-infected dogs in Brazil, (ii) to compare the sensitivity of rK39 RDT with crude antigen ELISA, which was a highly sensitive test in this cohort [3], [31], (iii) to examine variation in sensitivity during the course of infection, and in dogs with varying parasite loads and antibody titres to crude parasite antigen, and (iv) to use meta-analytical techniques to estimate the combined sensitivity and specificity of rK39 RDTs from published studies. The first three aims take advantage of samples from a very well-characterized cohort of dogs in Brazil, which were sampled bimonthly during the acquisition and development of natural infection and infectiousness in Amazon Brazil [3], [32].

## Methods

### Ethics statement

Canine samples were collected with informed consent from dog owners. Sampling was performed in accordance with UK Home Office guidelines.

### Study site and study design

Serum samples were selected using archived material from a prospective cohort study carried out from April 1993 to July 1995 in the municipality of Salvaterra, Marajó Island, Pará State, Brazil. The study design has been described previously [3], [31], [32]. Briefly, 126 initially uninfected dogs were placed at intervals within households in the study site, and sampled approximately every 2 months (mean interval 67 days, range 58–80 days) during exposure to natural infection, for a maximum of 27 months. Of the 86 dogs that became infected during the study, we selected 322 samples from the 54 dogs with the longest periods of follow-up after infection. Time of patent infection in study dogs was defined using our previous results as the first time point of detection of *Leishmania* infection by any of the following methods: (i) detection of anti-*Leishmania* IgG by ELISA using crude leishmanial antigen (CLA), with antibody concentrations expressed as arbitrary units/mL relative to a positive control serum (n = 322) [31]; (ii) PCR on bone marrow biopsies using primers specific for kinetoplast DNA (kDNA) and ribosomal RNA (n = 196) [31]; (iii) quantitative kDNA PCR on bone marrow biopsies, with results expressed as parasites/mL (n = 151) [6]; (iv) rK39 ELISA, with antibody concentrations expressed as signal/positive (s/p) ratio (n = 179) [33], where the cut-off was calculated from the back-transformed mean +3 SD of the log_10_ s/p ratios of 12 endemic control dogs. All samples taken on or after the time of patent infection were classified as from an infected dog. Dogs were also clinically examined at each time point, and assigned a semi-quantitative clinical score by scoring on a scale 0 (absent) to 3 (intense) six typical clinical signs of leishmaniasis (alopecia, dermatitis, chancres, conjunctivitis, onychogryphosis, and lymphadenopathy) (n = 295) [31]. A proportion of dogs was also assessed for infectiousness to the sandfly vector by xenodiagnosis, using uninfected colony-reared *Lutzomyia longipalpis* (n = 122) [3]. Negative control dogs comprised (i) 30 unexposed, non-endemic UK dogs with no history of foreign travel that had attended two UK veterinary clinics during June to December 2007, (ii) 8 non-endemic control samples from Brazilian study dogs prior to being placed in the endemic area, and (iii) 29 endemic control samples from 28 Brazilian study dogs taken prior to infection.

### Sample storage and quality control

Serum samples were collected during 1993–1995 and aliquotted at the time of collection. For long-term storage, samples were kept at −80°C. CLA ELISA was carried out in 1996, and rK39 ELISA and RDTs in 2008. Samples had been briefly thawed up to 5 times by the time of rK39 testing. Prior to the use of rK39 RDTs, all samples (n = 180) tested by rK39 ELISA were also re-tested by CLA ELISA to ensure continued sero-reactivity. A single sample showed reduced reactivity and was removed from further analysis. The remaining samples showed a good agreement with the results of the initial CLA ELISA, with a strong and consistent positive correlation between antibody concentrations in 1996 and 2008 (r^2^ = 0.78). 127/179 (71%) samples were seropositive in 1996 and 124/179 (69%) in 2008, with a high degree of concordance between years (kappa = 0.83) and no significant difference in sensitivity (McNemar's χ^2^ test, P = 0.41).

### rK39 RDT

rK39 RDTs (Kalazar Detect Canine Rapid Test: Lot No. HA1047 and HD1037) were obtained from Inbios International Inc., WA, USA, and used to test 20 µL serum samples according to the manufacturer's instructions. The appearance of a red line in the test area, however faint, was interpreted as positive following manufacturer's recommendations; all samples tested were classified as either positive or negative. All RDTs were performed by the same person, who was not blind to the results of reference tests.

### Meta-analysis

Literature searches were carried out in Medline and Web of Knowledge for articles published from 1993 to the end of 2011 using the keywords leishman* AND (canine OR dog) AND (rK39 OR K39 OR dipstick OR immunochromatographic OR ICT OR strip OR ‘rapid test’ OR ‘rapid diagnostic test’ OR RDT OR Trald). This search produced 100 papers, for which the titles, abstracts and, if necessary, full text were examined. Inclusion criteria for studies were (i) original studies, published in full (not conference abstracts), (ii) studies of natural infection with *L. infantum* (including *L. chagasi*), (iii) index test using rK39 antigen only in a dipstick format, (iv) data presented on the sensitivity and/or specificity of the index test, or derivable from from the data presented, and (v) infection confirmed by a suitable reference test (serology, parasitology or PCR). All studies that met these criteria were included, regardless of study design, and including both studies of diagnostic accuracy and studies that used rK39 RDTs in other contexts, or that reported only sensitivity data. Data were extracted on the number of study animals, country in which the study was carried out, study design, supplier of the dipstick test, case and control definitions and test results (absolute numbers of true positives, false positives, true negatives and false negatives). Where the latter numbers were not directly reported, they were derived from the reported sample sizes and sensitivity, specificity or prevalence. Cases were recorded as (i) clinical cases, defined as dogs with one or more symptoms suggestive of ZVL, and infection confirmed by one or more diagnostic tests (serology, PCR or parasitology) (ii) asymptomatic cases, or (iii) all infected cases, with confirmed infection irrespective of clinical status. Controls were classified as (i) non-endemic controls (NEC: dogs living in areas with no *Leishmania* transmission), (ii) endemic controls (EC: dogs living in endemic areas but with negative diagnostic test result for *Leishmania* infection) and (iii) dogs with other diseases (OD: dogs with confirmed disease that could potentially cross-react serologically). Study design was recorded as case-control (separate defined groups of cases and controls), cross-sectional (animals from a cross-sectional survey divided into cases and controls by one or more diagnostic tests) and cohort. Where studies compared the performance of rK39 with other serological tests in dogs whose status was defined by parasitology/PCR rather than serology, results of these serological tests were also extracted in the same way. Results from the current study were included in the meta-analysis; to avoid inflation of the weighting of this study due to multiple sampling of individual dogs, the estimated number of positive dogs was calculated from the overall proportion of samples positive and the number of dogs sampled (Table 1). Where studies used two reference tests, cases were defined as positive in either test, and controls as negative in both tests. Two studies used DAT as a reference test and presented rK39 results stratified by DAT titre [16], [21]: in this case the cut-off titre was taken as 1∶320, and effects of using a different cut-off tested. One study [15] tested two formulations of rK39 dipsticks, using protein A or protein G, with very similar results – results for the protein A formulation only were included in the meta-analysis.

**Table 1:** Sensitivity of rK39 RDT and CLA ELISA in samples from infected and uninfected dogs.

	n (N)	CLA ELISA	rK39 RDT
		% (95% CI)	% (95% CI)
Infected dogs	285 (54)	85.3 (80.4–89.1)	45.6 (36.8–54.7)
Uninfected endemic dogs	29 (28)	-	0
Brazil non-endemic dogs	8 (8)	-	0
UK non-endemic dogs	30 (30)	-	0
Infected dogs by serological status^1^			
- seropositive	243 (54)	-	52.2 (42.1–62.0)
- seronegative	42 (32)	-	0
Infected dogs by PCR status			
- PCR positive	111 (45)	91.8 (85.0–95.6)	58.6 (47.8–68.7)
- PCR negative	74 (30)	67.7 (53.5–79.3)	26.3 (14.7–42.6)
Infected dogs by clinical status			
- Symptomatic	99 (39)	95.3 (83.9–98.8)	76.5 (63.8–85.7)
- Polysymptomatic	39 (25)	97.4 (82.6–99.7)	88.9 (70.2–96.5)
- Oligosymptomatic	60 (30)	93.4 (79.7–98.1)	69.1 (52.5–81.9)
- Asymptomatic	178 (51)	81.3 (75.0–86.4)	32.6 (23.9–42.7)
Infected dogs by xenodiagnosis			
- Infectious	35 (18)	100	77.7 (59.0–89.4)
- Non-infectious	81 (27)	80.6 (69.0–88.5)	46.6 (32.0–61.8)

The percentage of dogs positive (95% CI) by each test for different classes of dogs was estimated using general estimating equations with robust standard errors, to account for the non-independence of repeat samples from the same dog. n – number of samples; N – number of dogs.

1 as defined by CLA ELISA.

### Statistical analysis

*Cohort data* The proportions of dogs positive by CLA ELISA and rK39 RDT were estimated from generalized estimating equation logistic regression models to control for within-dog autocorrelation; 95% confidence intervals were estimated using robust standard errors. Similar models were used to test associations between rK39 RDT results and other dog variables (i.e. time since infection, clinical status, xenodiagnosis, CLA ELISA and PCR results). Test sensitivities were compared using McNemar's χ^2^ test. Analysis was carried out in Stata 11.1 (Stata Corporation, College Station, Texas, USA).

*Meta-analysis* Sensitivity and specificity were calculated for each study group, with Wilson 95% confidence intervals [34]. Meta-analysis was performed using bivariate random-effects logistic regression models, with study included as the random effect [35]. Analyses were performed in Stata 11.1, using the ‘metandi’ command, and the options ‘glamm’ and ‘force’ to include any studies that did not provide data on either sensitivity or specificity. Sensitivity was estimated for clinical cases, asymptomatic cases and all infected cases (studies including both clinical and asymptomatic cases). For an overall estimate of specificity, an additional analysis of all studies with specificity data was performed. Publication bias was assessed visually from plots of sensitivity and specificity in each study against the number of cases or controls.

## Results

### Cross-sectional analysis

A total of 133/322 (41%) serum samples from 38/54 (70%) Brazilian dogs tested rK39 RDT positive, while 16/54 (30%) dogs remained rK39 RDT negative throughout the course of sampling. By comparison, CLA ELISA gave positive results in 243/322 (75%) samples from 54/54 dogs. Using samples from dogs with confirmed infections (see Methods), the overall sensitivity for detection of infection by rK39 RDT was estimated as 46% (95% CI 37–55%)(Table 1). Sensitivity was higher (77%, 95% CI 64–86%) in symptomatic infected dogs, and there was a strong association between rK39 RDT positivity and disease severity: sensitivity increased from 33% (95% CI 24–43%) in asymptomatic dogs, to 69% (95% CI 53–82%) in oligosymptomatic and 89% (95% CI 70–97%) in polysymptomatic dogs (Table 1). Sensitivity was also higher in dogs that were infectious at the time of sampling (78%, 95% CI 59–89%) compared to non-infectious dogs (47%, 95% CI 32–62%). As previously reported, CLA ELISA was generally a highly sensitive test in this study population [31], and the sensitivity of CLA ELISA was significantly superior to rK39 RDT in all classes of dogs (P≤0.031) except for polysymptomatic dogs, in which sensitivity of the 2 tests did not differ significantly (P = 0.13). All 30 UK non-endemic dogs were negative by rK39 RDT, as were all 37 samples from 36 uninfected Brazilian dogs, giving a specificity of 100% to detect infection.

**Figure 1:**
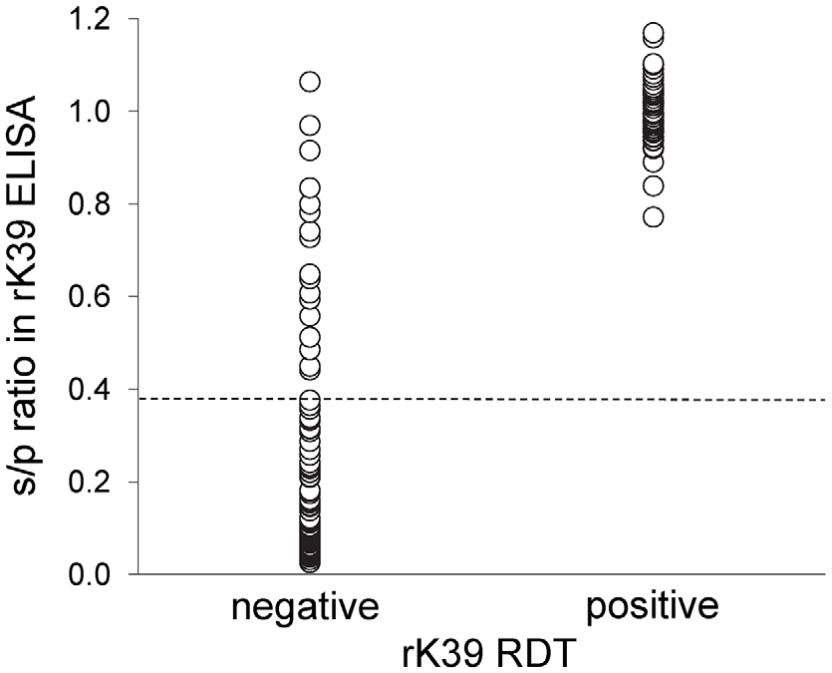
rK39 ELISA results in Brazilian dogs that tested positive or negative by rK39 RDT. rK39 ELISA results expressed as signal/positive (s/p) ratio. Dashed line indicates the rK39 ELISA cut-off.

Results were available from rK39 ELISA for a subset of 179/322 of the samples which were tested with rK39 RDT. As shown in Figure 1, there was a strong positive association between rK39 RDT positivity and the rK39 ELISA s/p ratio: no sample with s/p ratio <0.77 was RDT positive, whereas 92% (60/65) of samples above this point were RDT positive. In samples from dogs with confirmed infections tested by both methods, the sensitivity of rK39 RDT was 39% (60/155), lower than the sensitivity of rK39 ELISA (49%: 76/155; McNemar's χ^2^ = 16.0, P<0.0001). The sensitivity of rK39 ELISA was lower than that of CLA ELISA (83%, 128/155) in these samples, as previously reported [33].

### Longitudinal analysis

**Figure 2:**
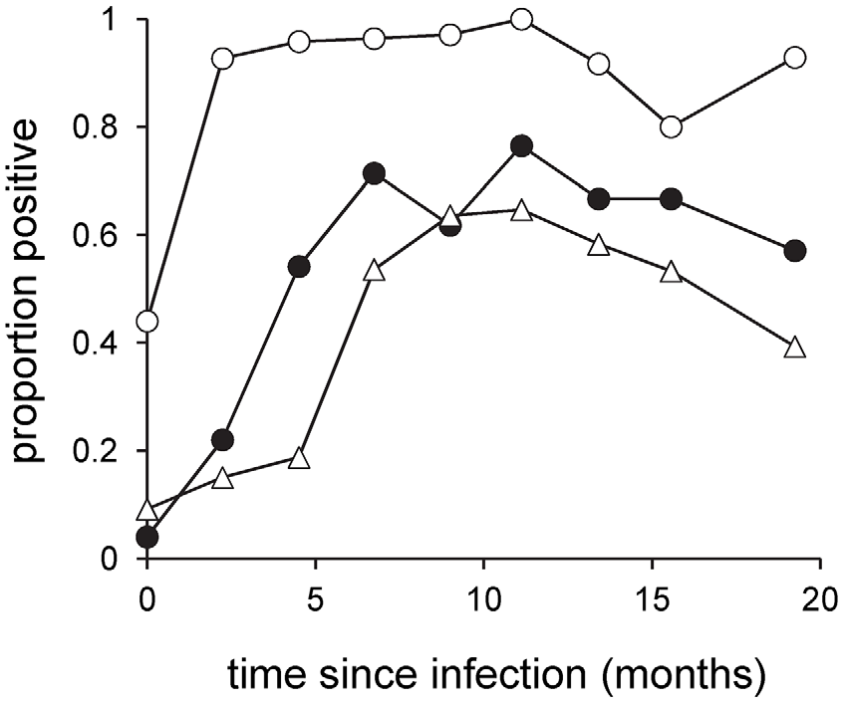
Sensitivity of CLA ELISA and rK39 RDT, and the proportion of symptomatic dogs, through time. Samples (n = 285) were aligned by time from first detection of infection. Key: CLA ELISA (open circles); rK39 RDT (closed circles); symptomatics (triangles). Each point represents 15–50 dogs.

rK39 RDT sensitivity increased with time since confirmed infection to a plateau by 6 months after infection; this was slower than the increase in CLA ELISA positivity, which reached maximal prevalences at 2–4 months after infection (Figure 2). The proportion of rK39 RDT positive dogs reached a maximum 2 months earlier than the proportion of symptomatic dogs (Figure 2). The relationship between rK39 RDT positivity and time from patent infection was highly significant and non-linear, with significant effects of both time and time^2^ (both P<0.0001)(Figure 2). There was no decline in the proportion of dogs that were RDT positive at later time points, indicating minimal serorecovery. This was confirmed by examination of test results for individual dogs: of 36 dogs which tested positive by rK39 RDT at >1 time point, only 3 (8.3%) dogs had a single sample that tested rK39 RDT negative after rK39 seroconversion (1/3 of which was borderline positive by CLA ELISA). In all cases the negative rK39 RDT result was followed by a positive result in the next sample.

**Figure 3:**
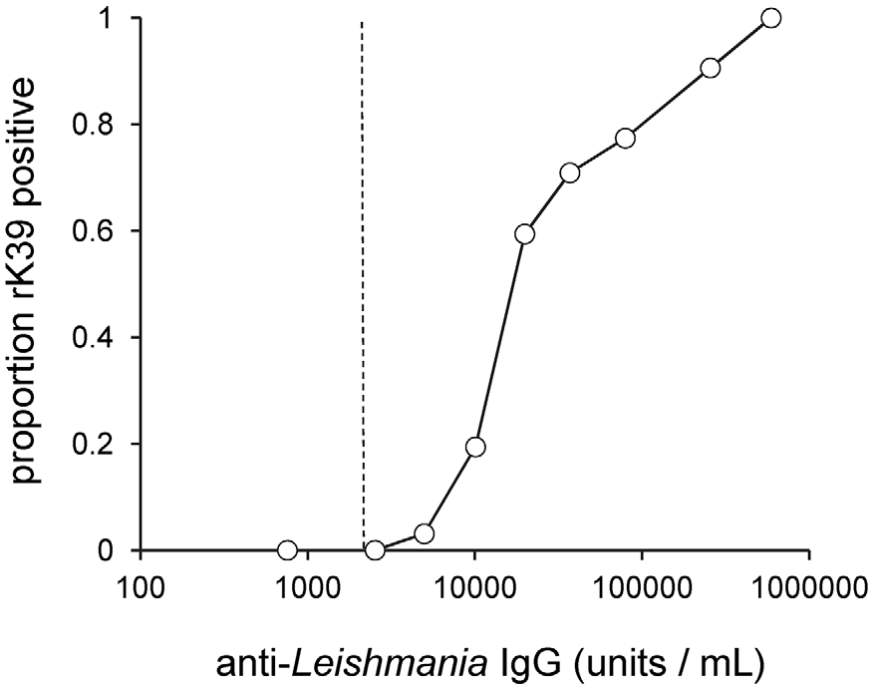
Proportion of dogs positive by rK39 RDT according to anti-CLA IgG concentrations. IgG concentrations expressed as arbitrary units/mL relative to positive control serum, by ELISA. Each point represents 31–32 samples. Dashed line indicates the CLA ELISA cut-off.

**Figure 4:**
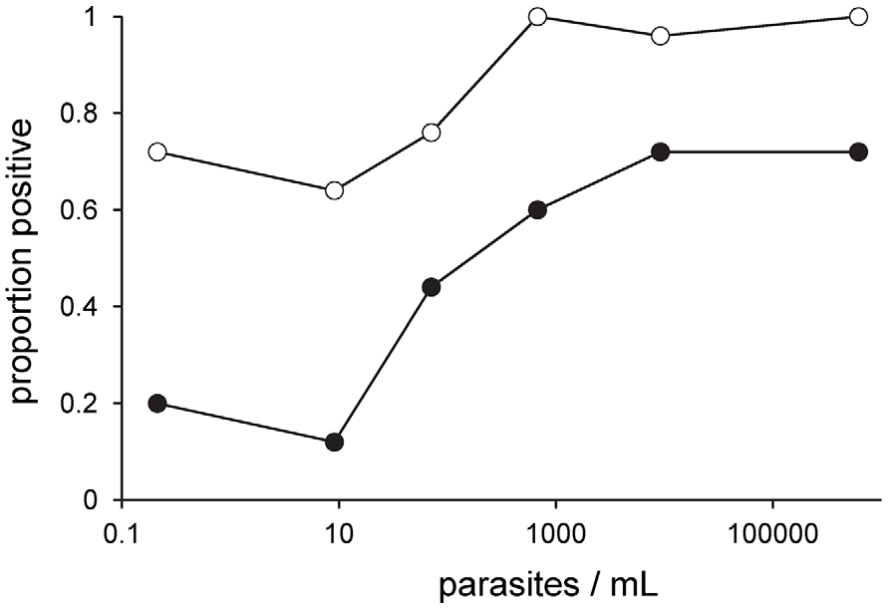
Proportion of infected dogs positive by CLA ELISA and rK39 RDT according to parasite density in bone marrow. Parasite density expressed as number of parasites/mL of bone marrow biopsy by quantitative kDNA PCR. Key: CLA ELISA (open circles); rK39 RDT (closed circles). Each point represents 25 samples.

RDT positivity in samples from infected dogs increased significantly with the log_10_ CLA ELISA titre, reaching 100% in dogs with the highest antibody concentrations (Table 2; Figure 3). Seropositivity in both CLA ELISA and rK39 RDT increased significantly with bone marrow parasite load, assessed by quantitative kDNA PCR, but rK39 RDT positivity only reached 72% even in dogs with the highest parasite loads (Table 2; Figure 4). The odds of being rK39 RDT positive were significantly higher in samples from dogs that were symptomatic (OR = 1.97, 95% CI 1.10–3.51), PCR positive (OR = 5.99, 95% CI 2.34–15.3) or infectious to sandflies (OR = 3.68, 95% CI 1.87–7.24) (Table 2). When adjusting analysis to include all explanatory variables, only high CLA ELISA titre and increasing time since infection remained as significant predictors of a positive rK39 RDT. Similar associations were seen between dog infection variables and rK39 ELISA results (data not shown).

**Table 2:** Factors associated with a positive rK39 RDT result in samples from infected Brazilian dogs.

Variable	Odds Ratio (95% CI)	*P*
PCR status: negative	1	
positive	5.99 (2.34–15.3)	0.0002
Clinical status: asymptomatic	1	
oligosymptomatic	1.47 (0.77–2.80)	
polysymptomatic	3.47 (1.47–8.17)	0.015
Clinical status: asymptomatic	1	
symptomatic	1.97 (1.10–3.51)	0.022
Infectious: no	1	
yes	3.68 (1.87–7.24)	0.0002
CLA ELISA titre (per 10× increase)	20.2 (8.52–48.0)	<0.0001
Parasite burden (per 10× increase)	1.64 (1.22–2.20)	0.001

Odds Ratios (95% CI) were estimated using general estimating equations with robust standard errors, to account for the non-independence of repeat samples from the same dog. All analyses included time since confirmed infection (months and months^2^) as covariates.

### Meta-analysis

**Figure 5:**
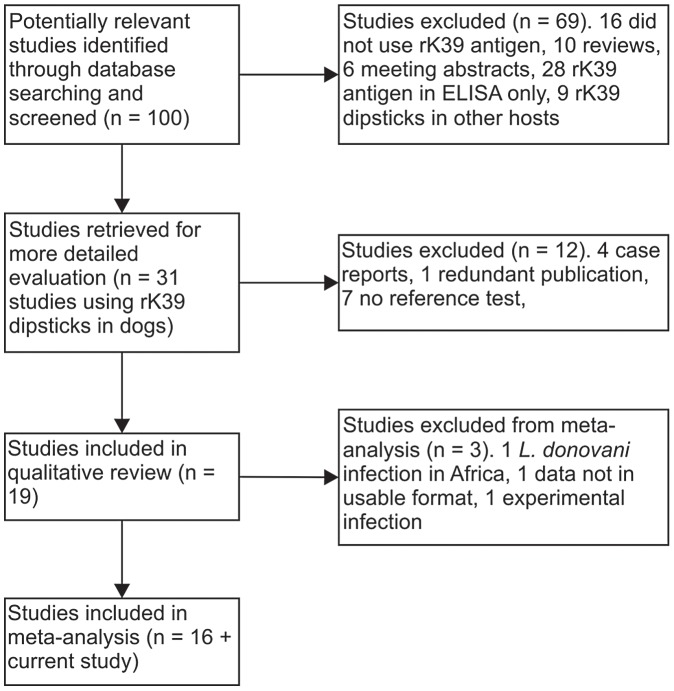
Flow diagram for study inclusion in meta-analysis.

The literature search retrieved 31 studies reporting the use of rK39 RDTs (dipsticks) in dogs, of which 16 original studies, plus the current study, reported data on the sensitivity of rK39 RDTs to detect canine infection (Figure 5). Eleven of these studies also included data on specificity. Of the 17 studies, 9 were carried out in the New World and 8 in the Old World; studies included 526 dogs with clinical disease (from 12 studies), 299 dogs with asymptomatic infection (from 7 studies), 431 dogs with confirmed infection (from 10 studies which included both clinical and asymptomatic dogs) and 1411 uninfected controls (from 11 studies). Twelve studies reported sensitivity in symptomatic infected dogs, selected on the basis of one or more clinical signs (Table 3). The estimated combined sensitivity in symptomatic dogs was 86.7% (95% CI 76.9–92.8%). Sensitivity varied from 50–100% in individual studies: sensitivity was high (85–97%) in 5 case-control studies, but lower in most cross-sectional studies. If a higher cut-off of 1∶640 was used for the one study with DAT as a reference test [16], the combined rK39 RDT sensitivity for symptomatics across all studies increased to 89.6% (95% CI 79.5–95.0%).

**Table 3:** Estimates of the sensitivity of rK39 RDTs to detect symptomatic visceral leishmaniasis in dogs.

Country	Study design^1^	Supplier^2^	Reference test	Number of cases	rK39 RDT % (95% CI)	ELISA^4^ %	IFAT %	Ref.
Brazil	CC	Inbios	IFAT	50	96.0 (86.5–98.9)	-	-	[15]
Iran	XS	Cypress	DAT	126	73.8 (65.5–80.7)	-	-	[16]
Turkey	XS	Inbios	Smear or IFAT	12	100 (75.8–100)	-	-	[17]
Mediterranean	CC	Diamed	Culture or PCR	30	96.7 (83.3–99.4)	100	90	[18]
Italy	CC	Intersep	Smear	68	97.1 (89.9–99.2)	-	98.5	[19]
Turkey	XS	Intersep	ELISA	8	87.5 (52.9–97.8)	-	100	[20]
Brazil	CC	Inbios	Smear	60	85.0 (73.9–91.9)	95	-	[22]
Brazil	XS	Inbios	ELISA	38	57.9 (42.2–72.1)	-	-	[23]
Argentina	XS	Inbios	PCR	42	66.7 (51.6–79.0)	-	66.7	[25]
Brazil	CC	-3	PCR	47	91.5 (80.1–96.6)	100	-	[26]
China	XS	Inbios	PCR	6	50.0 (18.8–81.2)	83.3	-	[28]
Brazil	Cohort	Inbios	PCR or ELISA	39	76.9 (61.7–87.4)	95	-	this study
Combined					86.7 (76.9–92.8)			

1 CC – case-control; XS – cross-sectional;

2 Inbios International Inc, WA, USA; Cypress Diagnostic Company, Belgium; Intersep DiaSys Europe Ltd; Diamed AG, Switzerland;

3 not stated;

4 crude antigen.

In contrast, the sensitivity of rK39 RDTs to detect asymptomatic infection was low, with a combined sensitivity of 49.7% (95% CI 29.3–70.1%) across 7 studies (Table 4). The sensitivity to detect all infected dogs irrespective of clinical status was slightly higher, 59.3% (95% CI 37.9–77.6%), though these results are not directly comparable due to the somewhat different sets of studies in each analysis (Table 5). Using a higher DAT cut-off in one study [21] increased the overall sensitivity of rK39 RDT to detect infected dogs to 63.9% (95% CI 42.0–81.2%). Sensitivity to detect asymptomatic or infected dogs was highly variable between studies.

**Table 4:** Estimates of the sensitivity of rK39 RDTs to detect asymptomatic *Leishmania infantum* infection in dogs.

Country	Study design^1^	Supplier^2^	Reference test	Number of dogs	rK39 RDT % (95% CI)	ELISA^4^ %	IFAT %	Ref.
Mediterranean	CC	Diamed	Culture or PCR	17	52.9 (31.0–73.8)	100	29	[18]
Turkey	XS	Intersep	ELISA	7	85.7 (48.7–97.4)	-	100	[20]
Brazil	CC	Inbios	Smear	16	75.0 (50.5–89.8)	94	-	[22]
Italy	XS^3^	Intersep	ELISA	143	55.9 (47.8–63.8)	-	59	[24]
Argentina	XS	Inbios	PCR	9	55.6 (26.7–81.1)	-	60	[25]
China	XS	Inbios	PCR or ELISA	56	10.7 (5.0–21.5)	61	-	[28]
Brazil	Cohort	Inbios	PCR or ELISA	51	33.3 (22.0–47.0)	81	-	this study
Combined					49.7 (29.3–70.1)			

1 CC – case-control; XS – cross-sectional;

2 Inbios International Inc, WA, USA; Cypress Diagnostic Company, Belgium; Intersep DiaSys Europe Ltd; Diamed AG, Switzerland;

3 data from first time-point of a cohort study;

4 crude antigen.

**Table 5:** Estimates of the sensitivity of rK39 RDTs to detect *Leishmania infantum* infection in dogs.

Country	Study design^1^	Supplier^2^	Reference test	Number of dogs	rK39 RDT % (95% CI)	ELISA^4^ %	IFAT %	Ref.
China	XS	Corixa	Smear	3	100 (43.9–100)	-	-	[13]
Brazil	XS	Intersep	PCR or ELISA	74	71.6 (60.5–80.6)	-	-	[14]
Mediterranean	CC	Diamed	Culture or PCR	47	80.9 (67.5–89.6)	100	68	[18]
Turkey	XS	Intersep	ELISA	15	86.7 (62.1–96.3)	-	100	[20]
Iran	XS	Cypress	DAT	26	38.5 (22.4–57.5)	-	-	[21]
Brazil	CC	Inbios	Smear	76	82.9 (72.9–89.7)	95	-	[22]
Argentina	XS	Inbios	PCR	51	64.7 (51.0–76.4)	-	65.4	[25]
Brazil	XS^3^	Inbios	IFAT	23	13.0 (4.5–32.1)	-	-	[27]
China	XS	Inbios	PCR or ELISA	62	14.5 (7.8–25.3)	59	-	[28]
Brazil	Cohort	Inbios	PCR or ELISA	54	46.3 (33.7–59.4)	85	-	this study
Combined					59.3 (37.9–77.6)			

1 CC – case-control; XS – cross-sectional;

2 Inbios International Inc, WA, USA; Corixa, USA; Cypress Diagnostic Company, Belgium; Intersep DiaSys Europe Ltd; Diamed AG, Switzerland;

3 data from first time-point of a cohort study;

4 crude antigen.

Considering all meta-analyses, there were no clear geographical differences in sensitivity between studies, though one Chinese study reported notably low sensitivity [28]. An additional study of *L. donovani* infection (thus not included in the meta-analysis) reported a low sensitivity (31%) of rK39 RDT to detect infection in Ethiopian dogs [36]. Of the 17 studies, 8 used tests obtained from Inbios International Inc, and 6 used tests distributed by Cypress Diagnostic Company, Intersep or Diasys Europe Ltd that were presumed to be tests manufactured by Inbios. Only one study used a test from a different manufacturer, Diamed AG [18], while two studies did not provide manufacturer details [13], [26]. Comparisons between tests from different manufacturers were thus not possible. In studies that directly compared the sensitivity of rK39 RDTs with crude antigen ELISA or IFAT, ELISA was more sensitive in all comparisons, while sensitivity of IFAT was comparable to that of dipsticks (Tables 3, 4, 5). Only one study reported a higher sensitivity of rK39 RDT (42%) compared to both ELISA (12%) and IFAT (18%) [37]; data from this study were not presented in a suitable format for inclusion in the meta-analysis. Studies used a variety of reference tests to confirm infection (Tables 3, 4, 5). Generally higher sensitivities were reported by the few studies that used parasitological confirmation (examination of smears or culture), compared to serology or PCR as a reference test.

The estimated combined specificity of rK39 RDTs was 98.7% (95% CI 89.5–99.9%). There was high specificity (93–100%) in 8/11 studies, but much lower specificity (65–85%) in 3 studies (Table 6). This variation did not appear to be related to the type of control used, but seemed to be study-specific. Specificity using groups of dogs with other confirmed infections was comparable to that using endemic and non-endemic control dogs (Table 7). Cross-reactions using RDTs were tested in few studies, but included dogs infected with *Ehrlichia canis* (1 of 3 dogs), *Trypanosoma cruzi* (3/12) and *Neospora caninum* (1/9) [18], [22]. There was no evidence for publication bias in either sensitivity or specificity.

**Table 6:** Estimates of the specificity of rK39 RDTs in dogs.

Country	Study design^1^	Supplier^2^	Reference test	Number and type of controls^3^	Specificity % (95% CI)	Ref.
Brazil	XS	Intersep	PCR/ELISA	40 NEC, 101 EC	65.2 (57.1–72.6)	[14]
Brazil	CC	Inbios	IFAT	50 NEC, 14 OD	100 (94.3–100)	[15]
Iran	XS	Cypress	DAT	152 EC	84.9 (78.3–89.7)	[16]
Turkey	XS	Inbios	Smear/IFAT	10 EC^6^	100 (72.2–100)	[17]
Mediterranean	CC	Diamed	-5	50 NEC, 26 OD	94.7 (87.2–97.9)	[18]
Italy	CC	Intersep	Smear	22 NEC, 33 EC, 42 OD	100 (96.2–100)	[19]
Brazil	CC	Inbios	Smear/ELISA	33 NEC, 25 OD	93.1 (83.6–97.3)	[22]
Brazil	XS	Inbios	ELISA	9 EC^6^	77.8 (45.3–93.7)	[23]
Italy	XS^4^	Intersep	ELISA	694 EC	100 (99.4–100)	[24]
China	XS	Inbios	PCR/ELISA	44 EC	97.7 (88.2–99.6)	[28]
Brazil	Cohort	Inbios	PCR/ELISA	38 NEC, 28 EC	100 (94.5–100)	this study
Combined					98.7 (89.5–99.9)	

1 CC – case-control; XS – cross-sectional;

2 Inbios International Inc, WA, USA; Cypress Diagnostic Company, Belgium; Intersep DiaSys Europe Ltd; Diamed AG, Switzerland;

3 NEC non-endemic controls, EC endemic controls, OD other diseases;

4 data from first time-point of a cohort study;

5 not stated;

6 clinically suspect dogs.

**Table 7:** Specificity of rK39 RDTs in various groups of control dogs.

Reference	Non-endemic (NEC)	Endemic (EC)	Other diseases (OD)
[14]	75.0	61.4	-
[15]	100	-	100
[16]	-	84.8	-
[17]	-	100	-
[18]	94.0	-	96.2
[19]	100	100	100
[22]	100	-	84.0
[23]	-	77.8	-
[24]	-	100	-
[28]	-	97.7	-
This study	100	100	-

## Discussion

The present study is the most detailed study to date of the factors affecting rK39 RDT positivity in dogs. The overall sensitivity of the rK39 RDT in all samples from infected dogs in the Brazil cohort was low (46%). Sensitivity to detect symptomatic dogs was higher (77%). Previous studies employing the rK39 antigen have also shown an association between rK39 positive test results and presence of active clinical disease in both humans [11] and in dogs [18], [22], [38]–[40]. Our results show that antibody responses to rK39 in natural infection develop more slowly than responses to crude antigen: dipstick sensitivity was particularly low in the early stages of infection and increased to a maximum 6–8 months after patent infection. Anti-rK39 responses developed before the peak of clinical infection, as also observed in experimental infection [30]. A positive RDT result was strongly associated with severe infection, being positively correlated with markers of disease progression, including parasite load, anti-*Leishmania* antibody level and time since infection. However, the proportion of RDT positive dogs did not reach 100% even in polysymptomatic dogs and dogs with the highest parasite loads (most likely to be responsible for the majority of transmission). The strongest association was between rK39 positivity and antibody titre, as reported in other studies [16], [21]. Infectiousness to sandflies is also a characteristic of dogs with severe disease [3]. The sensitivity of rK39 RDTs to detect infectious dogs has not previously been reported; here we found that infectious dogs (by xenodiagnosis) were more likely to be RDT positive, which is not unexpected given the associations between infectiousness, clinical symptoms and antibody concentrations [1], [3]. For intervention programmes, high sensitivity to detect infectious dogs is needed; ideally, tests would also be able to discriminate between infectious and non-infectious dogs, enabling the possibility of targeted control. However, the sensitivity of rK39 RDTs to detect infectious animals was only 78%, which is likely to be too low for an effective intervention programme [3].

Meta-analysis of published data, including data from the current study, suggests that rK39 RDTs provide a reasonably sensitive and specific test for infection in dogs with clinical symptoms. The overall sensitivity and specificity using a random-effects model were 87% (95% CI 77–93%) and 99% (95% CI 90–100%). The rK39 RDT sensitivity was slightly lower than the sensitivity (94%) in human clinical infection with *L. donovani* or *L. infantum* worldwide [12], but within the range reported for human *L. infantum* infection in Brazil (85–92%) [41]. Both the sensitivity and specificity varied across studies. The reasons for this variability are unknown, and numbers of studies are too small for a detailed comparison of different tests and geographical areas. Direct comparisons of the performance of different RDTs on the same samples are not available for canine infection; a recent multi-centre comparison of two rK39 RDTs on human samples suggested slight differences in performance [41]. Some geographical variation in RDT sensitivity for diagnosis of human VL has been reported, with highest sensitivity for *L. donovani* infections in the Indian subcontinent, and lower sensitivity in East Africa [41], [42], but geographical variation in sensitivity for human *L. infantum* infection has not been examined. Other possible explanations for the variation in sensitivity between canine studies include differences in the average severity of clinical signs, study-specific differences (e.g. in the methods to measure and define symptoms), and differences in the specificity of clinical diagnosis between regions. The specificity of clinical diagnosis is likely to be higher in Europe, compared to areas with a higher prevalence of other infections with similar symptoms. In addition, there was a trend towards higher sensitivity in studies that used parasitology (smears or culture) to confirm infection, which may reflect higher parasite burdens in these dogs compared to dogs positive by serology or PCR. The variability seen across studies highlights the need to develop standardized protocols for diagnostic tests in canine leishmaniasis, including standardized definitions of clinical severity (e.g. [8]), to perform differential diagnosis, and to carry out multi-centre trials of existing and novel tests.

Diagnostic tests using defined antigens typically have higher specificity than those using crude antigens, due to the lower cross-reactivity with other pathogens likely to be co-circulating in endemic areas affected by ZVL. The overall specificity of rK39 RDTs in dogs was indeed very high (99%), slightly higher than that reported in humans [12], [41]. However, 3 of 11 studies reported much lower specificities of 65–85% [14], [16], [23]. Specificity might be expected to be highest in healthy non-endemic controls, compared to healthy endemic controls (a proportion of which may have unapparent infection) or controls infected with other diseases (which may have cross-reacting antibodies). However, specificity in this analysis did not vary greatly according to the type of negative control, and most variation was seen within rather than between types of controls. Specificity in dogs with other diseases was not lower than in other groups of controls, as also reported for rK39 RDTs in humans [12]. A few false positive rK39 RDT results were reported in dogs with *Ehrlichia canis*, *Trypanosoma cruzi* or *Neospora caninum* infection [18], [22], though few studies tested dogs with potentially cross-reacting infections. rK39 is restricted to species of the visceralizing *L. donovani* complex [10], but cross-reactions have been reported using rK39 ELISA in 3/9 dogs infected with *L. braziliensis* [40], and using the rk39 RDT in two Iranian dogs infected with *L. tropica* [43], [44]. More extensive characterization of rK39 antigen specificity is required before recommendations can be made for the use of these tests in areas with such co-circulating pathogens.

In contrast to the high sensitivity of rK39 RDTs for *clinical disease*, the sensitivity to detect *canine infection* is much lower. The combined sensitivity to detect infection was only 59%, attributable to the low sensitivity to detect asymptomatic infection (50%), though variation in sensitivity between individual studies was high. The implications of the low overall sensitivity to detect infection are that rK39 RDTs are not an effective tool for estimating the prevalence of infection in epidemiological studies, nor for the identification of infected dogs in control programmes. In studies that directly compared the sensitivity of rK39 RDTs to crude antigen ELISA or IFAT to detect infection, the sensitivity of ELISA was generally higher while that of IFAT was low and similar to the RDT. The low sensitivity of rK39 RDTs to detect infection could be attributable to use of a single defined antigen, and/or to the dipstick format. In the current study, the sensitivity of rK39 ELISA was somewhat higher than rK39 RDTs, but much lower than that of CLA ELISA. Similarly, in a European study the sensitivity of CLA ELISA (100%) was much higher than either rK39 ELISA (83%) or rK39 RDT (81%) [18]. These results suggest that low sensitivity to detect asymptomatic infection is largely due to the use of a single defined antigen. The use of multiple defined antigens in ELISA has been shown to increase sensitivity, with the relative sensitivity of different antigens varying between dogs [39], [40], [45]–[47]. Thus the development of RDTs using multiple defined antigens, or crude antigens, may increase sensitivity [48], [49]. Nonetheless, recent tests of a RDT incorporating both rK26 and rK39 reported low sensitivity (47%) to detect asymptomatic canine infections [49].

In a veterinary clinical setting, the high specificity of rK39 RDTs indicates that they are a useful rapid test for diagnosis of clinically symptomatic cases of canine leishmaniasis, which currently is a diagnostic challenge as the clinical signs are not necessarily specific. However, additional diagnostic tests are needed for confirmation [8], particularly in clinically suspect, rK39 dipstick-negative animals, given the potential for false negative RDT results. In contrast, the sensitivity of rK39 RDTs to detect infected or infectious dogs is too low for their effective use in epidemiological studies or operational control programmes. The rapid availability of results from on-site dipstick testing compared to centralized laboratory serological tests is clearly desirable, as this would prevent the long delays between sampling and diagnosis which have been identified as an important reason for failure of dog culling to reduce human ZVL incidence in Brazil [3]. There may also be significant improvements in owner compliance when presented with an unequivocal, visually interpretable result at the time of testing. Further research is needed to develop more sensitive RDTs, using combinations of antigens.

## Supporting Information

Checklist S1 **STARD checklist.**
(DOC)

Checklist S2 **PRISMA checklist.**
(DOC)
